# Farnesol-induced hyperbranched morphology with short hyphae and bulbous tips of *Coriolus versicolor*

**DOI:** 10.1038/s41598-018-33435-6

**Published:** 2018-10-12

**Authors:** Ke-Feng Wang, Chen Guo, Fang Ju, Nadia A. Samak, Guo-Qiang Zhuang, Chun-Zhao Liu

**Affiliations:** 1 State Key Laboratory of Biochemical Engineering, Institute of Process Engineering, Chinese Academy of Sciences, Beijing, 100190 P.R. China; 2 University of Chinese Academy of Sciences, Beijing, 100049 P.R. China; 3 Department of Oncology, Qingdao Central Hospital, Qingdao, 266042 P.R. China; 4 Research Center for Eco-Environmental Sciences, Chinese Academy of Sciences, Beijing, 100085 P.R. China; 5 Institute of Biochemical Engineering, Collaborative Innovation Center for Marine Biomass Fibers, Materials and Textiles of Shandong Province, School of Materials Science and Engineering, Qingdao University, Qingdao, 266071 P.R. China

## Abstract

As the first fungal quorum sensing molecule, farnesol-induced morphological transition is usually studied in dimorphic fungi, but in basidiomycetes the morphological changes regulated by farnesol are rarely investigated. In this study, we found that farnesol made the basidiomycete *Coriolus versicolor* develop into a hyperbranched morphology with short hyphae and bulbous tips. Farnesol treatment resulted in a significant increase of intracellular oxidative stress level, which influenced the expression of several morphogenesis-related genes, and thereby led to the morphological changes. High oxidative stress level significantly stimulated the expression of laccase genes for improving intracellular laccase biosynthesis. The resulted hyperbranched morphology further accelerated the secretion of intracellular laccase into culture medium. As a result, extracellular laccase production reached a maximum of 2189.2 ± 54.7 U/L in farnesol-induced cultures, which was 6.8-fold greater than that of control cultures. SDS-PAGE and native-PAGE showed that farnesol increased laccase production by promoting the biosynthesis of three laccase isoforms. Together these results provide new opportunities in not only understanding the farnesol-regulated mycelial morphology in basidiomycetes, but also developing novel strategies for enhancing the production of secreted enzymes of biotechnological interest.

## Introduction

In filamentous fungi, mycelial morphology affects the synthesis and secretion of intracellular metabolites. It has become a key factor for the production of secretory protein^1^. Efficient secretion of intracellular protein can relieve the endoplasmic reticulum stress caused by mass-produced target protein, and promote its correct expression and processing. Fungal mycelial morphology directly restricts the production efficiency of target protein^2^. The application of morphology engineering in the production of secretory enzymes has become a research focus^1^. Some studies show that the ability of secreting extracellular enzyme can be considerably improved by changing the mycelial morphology. For example, bulbous hyphal tip of *Neurospora crassa mcb* mutant resulted from strictly temperature shift led to a three- to fivefold increase in the level of extracellular protein^3^. Freely dispersed mycelium caused by the addition of talc microparticle resulted in a fourfold increase of fructofuranosidase production in *Aspergillus niger*^4^. Bio-pellet morphologies of *A*. *niger* was developed by the addition of titanate microparticle (TiSiO_4_) to culture medium, which led to a 9.5-fold increase of glucoamylase level^5^. The regulation of mycelial morphology is an effective strategy to improve the production of secreted enzymes of biotechnological interest^1,6^.

Several studies have suggested that quorum sensing molecule (QSM) can induce complex cellular behavior including morphological switches and secretion of extracellular enzymes^7–9^. Farnesol as the first QSM in fungi is well characterized in *Candida albicans* where farnesol blocks the yeast-to-mycelium shift^10^. The morphological transition is regulated by diverse signal transduction pathways and transcriptional regulators including high osmolarity glycerol mitogen-activated protein kinase (Hog1 MAPK) pathway and filament-repressor Tup1^8,11^. Some researchers find that farnesol affects mycelial morphology of *Aspergillus fumigatus* by regulating Rho GTPase that control fungal morphogenesis, among which RhoA, RacA and CftA are three most extensively characterized members^12,13^. Farnesol also has other physiological effects, such as inhibiting biofilm formation, modulating drug efflux, inducing mass synthesis of reactive oxygen species (ROS) and increasing oxidative stress level^14,15^. In *Aspergillus nidulans*, the addition of farnesol to the cultures triggers morphological characteristics of apoptosis^16^. But in white-rot basidiomycetes the cells can initiate protection mechanism against oxidative stress, such as expressing large amounts of antioxidant enzymes^17^.

*Coriolus versicolor* as a white-rot basidiomycete degrading lignin has developed oxidative stress defense mechanisms^18^. Laccase (benzenediol: oxygen oxidoreductase, EC 1.10.3.2), an important ligninolytic enzyme, acts as an important element of the general stress response systems^17^. Laccase is capable of catalyzing the oxidation of a wide variety of phenolic compounds concomitantly with O_2_ reduction to H_2_O^19,20^. As an environment-friendly green catalyst, laccase possesses potentials and advantages of great interest for various biotechnology applications including pulp bleaching, wastewater treatment, biopolymer modification, biosensors and bioremediation of environmental pollutants^21,22^. In order to meet the high demand for laccase, it is necessary to increase laccase production. A few strategies have been developed to enhance laccase production, such as the screening of high-yield strains, heterologous expression of laccase genes, ultrasonic stimulation, and the utilization of inducers^23–25^. The commonly used inducers are metal ions and aromatic compounds^23^. In our previous study, we found that 60 μM farnesol stimulated laccase production by inducing a more branched mycelia of *C*. *versicolor*, but the induction mechanism was not fully elucidated^26^.

The objective of the current study is to understand the mechanism that farnesol induced the morphological changes related to physiological status of *C*. *versicolor*. Furthermore, the effect of these farnesol-induced changes on extracellular laccase production was investigated.

## Materials and Methods

### Microorganisms and chemicals

*Coriolus versicolor* was obtained from China Center of Industrial Culture Collection. This strain was subcultured every 2–3 weeks on potato dextrose agar (PDA) slants, grown at 26 °C and maintained in the refrigerator at 4 °C. Farnesol (mixture of isomers, F203) was obtained from Sigma–Aldrich (USA). The inductive effect of *E*,*E*-farnesol and farnesol (mixture of isomers) on laccase production was almost the same. So the farnesol (mixture of isomers) was used as the inducer for further experiment. All other chemicals were analytical grade and purchased from Beijing Chemical Reagents Company (Beijing, China).

### Culture media and growth conditions

*C*. *versicolor* mycelia were incubated on PDA (potato 200 g/L, dextrose 20 g/L, agar 15 g/L) slants at 26 °C. After one week, three mycelia plugs (~0.5 × 1.0 cm) were excised from the slant and inoculated into 250 mL Erlenmeyer flasks containing 100 mL of freshly prepared potato dextrose broth (potato 200 g/L, dextrose 20 g/L). Fungi were precultured for 7 days and the precultured broth was gently homogenized. Approximately 10 mL of the homogenate was transferred into 100 ml of potato dextrose broth in a 250 mL culture flask. After being cultured for 5 days, *C*. *versicolor* grew as 1 mm pellets and that 5 ml of the mycelial suspension, used as the seed for laccase production, was introduced into a 250 mL flask containing 50 mL of liquid growth medium. The liquid medium for laccase production consisted of (per liter of distilled water): 0.2 g of KH_2_PO_4_, 0.1 g of CaCl_2_⋅2H_2_O, 0.05 g of MgSO_4_⋅7H_2_O, 0.5 g of NH_4_H_2_PO_4_, 0.035 g of FeSO_4_⋅7H_2_O, 2 g of glucose, 0.27 g of NH_4_Cl, 0.02 g of CuSO_4_⋅5H_2_O. Before inoculation, farnesol was sterilized with filter membrane (0.22 μm, Millipore) and directly added to the cultures at a final concentration of 4.0 mM. The group without farnesol treatment was inoculated under the same conditions as the control. The pH of fermentation broth for laccase production was maintained at 4.0 with 1 mol/L HCl or NaOH during the culture process. All the cultures were incubated on a HYG-A double-deck TAT rotary shaker at 150 rpm, 26 °C. Culture flasks were sealed with sterile ventilated sealing film. The final approximate diameter of mycelial pellets was ~4.0 mm. Samples were taken periodically for 7 days from three replicate flasks, and all experiments were carried out in triplicates.

The liquid medium for laccase production from *Pycnoporus sanguineus* consisted of: 10 g L^−1^ glucose, 0.2 g L^−1^ ammonium tartrate, 0.5 g L^−1^ MgSO_4_⋅7H_2_O, 2 g L^−1^ KH_2_PO_4_, 0.1 g L^−1^ CaCl_2_⋅2H_2_O, 1 mg L^−1^ vitamin B_1_, 20 mM sodium acetate buffer (pH 4.5), 0.02% of a Hutner solution of trace elements (0.22 g of ZnSO_4_, 0.11 g of H_3_BO_3_, 0.05 g of MnCl_2_, 0.05 g of FeSO_4_, 0.016 g of CoCl_2_, 0.016 g of CuSO_4_, and 0.011 g of (NH_4_)_6_MO_7_O_24_ per 100 mL). The growth medium for *Trametes pubescens* laccase production consisted of: 20 g L^−1^ glucose, 5 g L^−1^ yeast extract, 5 g L^−1^ peptone from casein, 1 g L^−1^ KH_2_PO_4_, 0.5 g L^−1^ MgSO_4_⋅7H_2_O, 0.05 g L^−1^ ZnSO_4_⋅7H_2_O, 0.05 g L^−1^ CuSO_4_⋅5H_2_O. The pH value of two media was adjusted to 5.0 before sterilization. The other procedures of *P*. *sanguineus* and *T*. *pubescens* cultures were same as that of *C*. *versicolor*.

### Mycelial morphology evaluation

To observe the morphology, collected mycelial pellets were transferred to a petri dish with distilled water. Mycelial macromorphology was determined using a Canon camera and a stereo microscope (10×, Nikon SMZ1500). Culture micromorphology was examined using a light microscope (Leica Microsystems CMS GmbH, Germany) with 10×, 20× and 100× objectives. For SEM tests, mycelial pellets were immersed in 4% glutaraldehyde solution overnight. The fixed samples were then rinsed with a PBS solution (pH 7.0) for 3 times, followed by the dehydration in several stages with a serial dilution of ethanol (30%, 50%, 70%, 90% and 100% v/v) for 15 min each and further rinses in isoamyl acetate for more than 2 h. The samples were then critical-point- dried in CO_2_ for 3 h. Scanning electron microscopy (SEM) observations were performed at 10 kV with a Hitachi S3000N (Tokyo, Japan) according to the manufacturer’s instructions. To observe the growth of mycelium pellet treated with farnesol, the pellet of equal size was placed on the center of culture dish with PDA and grown at 26 °C. Mycelial growth was recorded using a Canon camera every day until the mycelia grew all over the plate. A group without farnesol treatment was carried out under the same conditions as the control.

### Analytical assays

Microbial biomass growth was determined by measuring the mycelia dry weight. After separation from the culture liquids by filtering, the pelleted mycelia were rinsed with distilled water and dried at 60 °C in a vacuum drying oven until they reached a constant weight. The cell-free supernatants were used to quantify laccase activity and extracellular protein concentration. Laccase activity was assayed spectrophotometrically using a reaction mixture containing 50 mM tartaric acid buffer (pH 4.0) with 0.1% (w/v) catechol as the substrate at 26 °C. A suitable amount of culture supernatants was added into the substrate solutions, and the increase in the absorbance at *A*_450_ (ε = 2,211 M^−1^ cm^−1^) was monitored. One unit of enzyme activity was defined as the amount of laccase required to oxidize 1 μmol of catechol per min^25,27^. The extracellular proteins were quantified with the Bradford Protein Assay Kit (Beyotime, China) according to the manufacturer’s instructions using bovine serum albumin as the standard. Extracellular laccase specific activity was expressed in U/mg-protein.

Sodium dodecyl sulfate–polyacrylamide gel electrophoresis (SDS-PAGE) of extracellular culture supernatants (20 uL) was carried out with a 12% cross-linked polyacrylamide gel according to Garcia’s methods^28^. Protein bands on the gel were visualized with silver staining and compared with a commercially available laccase standard from *C*. *versicolor* (Sigma–Aldrich). Native-PAGE of extracellular culture supernatants (20 uL) was performed at alkaline pH under non-denaturing conditions according to Pezzella’s methods^29^. Protein bands with laccase activity were visualized in 50 mM tartaric acid buffer (pH 4.0) with 0.1% (w/v) catechol. The gels of SDS-PAGE and Native-PAGE were scanned with a Molecular Imager (Bio-Rad Laboratories, USA).

For the determination of intracellular laccase activity in the mycelia, mycelial pellets were gathered from the fermentation broth by centrifugation for 5 min at 3000 × g and washed three times with distilled water. The mycelia were ground with silica sand and 100 mM sodium acetate buffer (pH 5.0, pre-cooled to 4 °C, the ratio of wet weight pellets (g) to buffer volume (ml) is 1:10) for 10 min in an ice bath, and then centrifuged at 10000 × g, 4 °C. Laccase activity in the supernatant was regarded as the intracellular fraction and was assayed as described above. Intracellular laccase activity was expressed in U/g-biomass. Intracellular ROS accumulation was detected using the fluorescent probe 2′,7′-dichlorofluorescein diacetate (DCFH-DA) (Beyotime, China) according to the manufacturer’s instructions with reactive oxygen species positive control (Rosup) as the standard. Intracellular oxidized glutathione (GSSG) was determined using GSH and GSSG Assay Kit (Beyotime, China) according to the manufacturer’s instructions.

### Quantification of the relative expression of morphogenesis-related genes and laccase genes

To evaluate the effect of farnesol on the relative expression of morphogenesis-related genes (*rhoA*, *racA*, *cftA*, *tup1* and *hog1*) and laccase genes (*lcc1*, *lcc2* and *lcc3*), fresh mycelia from triplicate cultures were collected from the culture broth by filtration, washed with distilled water, flash frozen in liquid nitrogen, and stored at −70 °C till use. Total RNA was extracted from the mycelia pellets with the Trizol^®^ Reagent (Invitrogen, USA) according to the manufacturer’s instructions. 1% agarose gel electrophoresis with formaldehyde was used to test the RNA integrity. The two bands of 28S rRNA and 18S rRNA were clear that indicated that the RNA was not degraded. Total RNA was quantified using spectrophotometer NanoDrop ND-2000. A260/A280 and A260/A230 were used to estimate the purity of RNA. The values of A260/A280 and A260/A230 were 1.9~2.0, which suggested that the purity of RNA was very high. Then RNA was treated with RQ1 RNase-Free DNase (Promega, USA) to get rid of genomic DNA according to the manufacturer’s instructions, and stored at −70 °C. Reverse transcription of RNA was performed using the GoScript^TM^ Reverse Transcription System (Promega, USA). 1 microgram of total RNA was used as the template to synthesize the cDNA with oligo (dT)_15_ primer according to the manufacturer’s instructions.

To carry out real-time quantitative PCR (qPCR) reactions, genes homologous to those regulating mycelial morphology, coding for *rhoA*, *racA* and *cftA* in *A*. *niger* and *Ustilago maydis*^13,30^, *tup1* and *hog1* in *Ophiostoma piceae* and *C*. *albicans*^7,8^, were identified in the publicly available genomes of *C*. *versicolor* FP-101664^31^. A BLAST search against the NCBI database was performed for these genes based on highest homology. After the sequences were aligned, primers were designed in the conserved regions and listed in Supplementary Information-Table S1. The primers of *lcc1*, *lcc2*, *lcc3* and 18S rRNA gene were designed on the basis of the sequences of *lcc1* (GeneBank accession number X84683), *lcc2* (GeneBank accession number AB212732), *lcc3* (GeneBank accession number AB212733) and 18S rRNA gene (GeneBank accession number AY336751).

qPCRs were performed using 7500 Fast Real-Time PCR Detection System (Applied Biosystems) with GoTaq^®^ qPCR Master Mix (Promega, USA) according to the manufacturer’s instructions. The PCR thermal cycling conditions were as follows: GoTaq^®^ hot start polymerase activation at 95 °C for 2 min and 40 cycles at 95 °C for 15 s, an annealing step (different primer pairs corresponding to different optimum annealing temperature) for 30 s, and 72 °C for 30 s, followed by a denaturation step to check the absence of unspecific products or primer dimmers. Each reaction mixture was set to a final volume of 20 μL: 10 μL of GoTaq^®^ qPCR Master Mix, 2.4 μL of each primer (10 μM), 7.52 μL of nuclease-free water and 2 μL of template cDNA from three independent replicates of *C*. *versicolor* control cultures or farnesol-induced cultures. In all experiments, appropriate reverse transcription-negative controls and controls containing no template were subjected to the same procedure to detect any possible contamination. Each sample was amplified twice in every experiment. The PCR efficiencies for all the primer pairs were measured by performing a 10-fold serial dilution of positive-control template to generate a standard curve and by plotting the C_T_ (C_T_ indicates the cycle in which a target sequence is first detected) as a function of log_10_ of template. The relative abundance of mRNAs was normalized against the level of 18S rRNA gene amplification run on the same plate. Quantification is relative to the endogenous control gene by subtracting the C_T_ of the control gene from the C_T_ of the gene of interest. The relative gene expression levels were calculated by a mathematical model, which includes a correction for real-time PCR efficiency of the individual transcripts^32^.

### Statistical analysis

The data were analyzed using a one-way analysis of variance (ANOVA) followed by Student’s *t*-test with the SPSS for Windows software. *P*-value ≤ 0.05 was regarded as significant, and data were presented as means ± standard deviation of three replicates.

## Results and Discussion

### Effect of farnesol on mycelial morphology of *C*. *versicolor*

**Figure 1:**
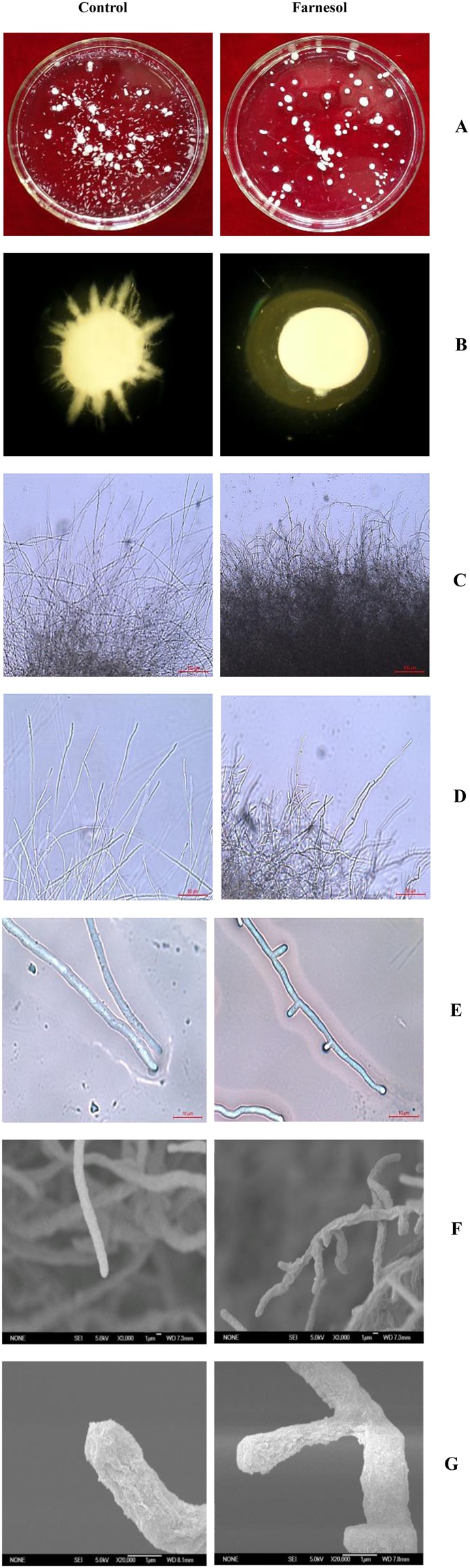
Morphological changes of *C*. *versicolor* at 96 h after inoculation in the presence and absence of farnesol (final concentration, 4.0 mM) in submerged cultures. Column A: macroscopic phenotype; Column B: microscopic phenotype with 10× magnification; Column C: microscopic phenotype with 100× magnification. Bar, 100 um; Column D: microscopic phenotype with 200× magnification. Bar, 50 um; Column E: microscopic phenotype with 1,000× magnification. Bar, 10 um; Column F: SEM tests with 3,000× magnification; Column G: SEM tests with 20,000× magnification.

The mycelial morphology of *C*. *versicolor* regulated by farnesol was observed in submerged processes (Fig. 1A–G). In farnesol-induced cultures, no long hyphae extended out from mycelium pellet compared with control cultures, creating smooth pellets (Fig. 1A,B). This allows for the pelleted growth that has been found to be beneficial for the production of secretory proteins in *A*. *niger*^33^. As shown in Fig. 1C–E, *C*. *versicolor* treated with farnesol had a higher branching frequency and displayed a hyperbranching phenotype (caused by dichotomous branching). The results confirmed our previous study that farnesol treatment resulted in a highly branched morphology of *C*. *versicolor*^26^.

**Figure 2:**
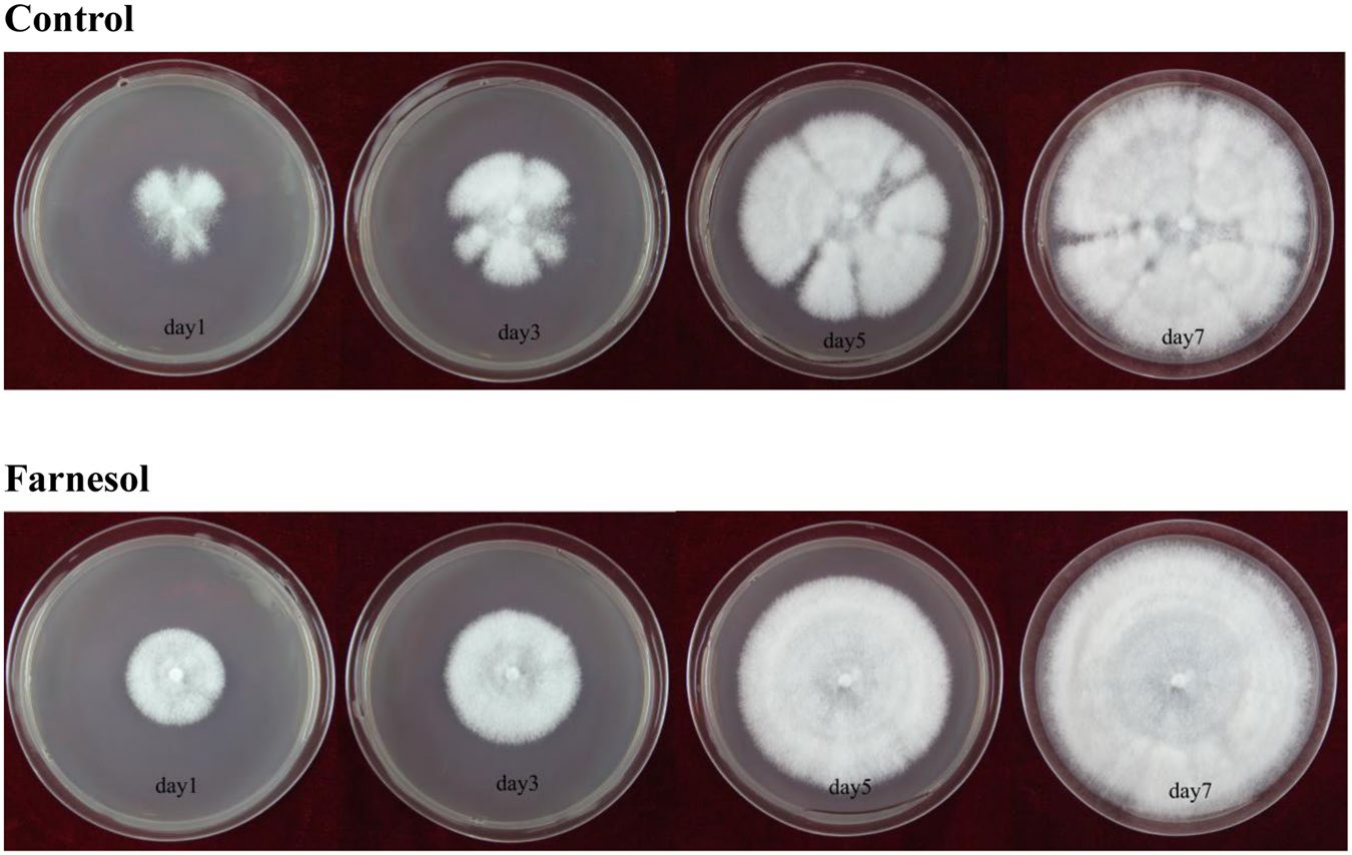
The growth of mycelium pellet treated (or not) with farnesol on culture dish with PDA.

More importantly, when the concentration of farnesol used was increased, *C*. *versicolor* not only showed the hyperbranched form, but also possessed short hyphae with bulbous tips. As shown in Fig. 1E,F, newly formed germ tubes and hyphae had a relatively narrow diameter in the highly branched *C*. *versicolor*. The length of parental hyphae and new daughter hyphae was comparatively shorter than that of the control. Similar phenomenon was also reported in *O*. *piceae* and *Penicillium decumbens* treated with farnesol^7,34^. Fig. 1G showed that new daughter hyphal tips of *C*. *versicolor* exposed to farnesol lost their copped morphology and formed a swollen-tip phenotype. The hyphae stopped extension growth and developed bulbous lateral branches along the parental hyphae, suggesting that farnesol provoked the loss of polarized tip extension. Meanwhile, the dichotomous branching phenotype indicated that the loss of apical dominance was overcome by the establishment of two new sites of polarized growth. The results were also confirmed by the growth phenotype of mycelium pellet on culture dish (Fig. 2). Mycelium pellet treated with farnesol presented homogeneous radial growth due to more hyphal tips that conducted the lateral growth continuously. Comparatively, the radial growth of mycelium pellet from control cultures was heterogeneous because the fluffy mycelium pellet had relatively less hyphal tips and undertook the longitudinal growth. The morphological characteristics were the same as that of *A*. *fumigatus* treated with farnesol^12^.

It is know that hyphal tip is the growth site in filamentous fungi, and protein secretion is mainly restricted to the tip known as Spitzenkorper^1,2^. More branching hyphae represented more active tips. Moreover, the hyphae were short and the active tips were swollen bulbous that suggested that the surface areas of protein secretion sites were increased. Lee *et al*. reported that increased surface areas of hyphal tips in *N*. *crassa mcb* mutant that showed a loss of growth polarity led to a significant increase in the amount of protein secretion and a 20-fold increase in carboxymethyl cellulase activity^3^. In general, farnesol made *C*. *versicolor* develop into a hyperbranched mycelial morphology with short hyphae and bulbous tips. This morphology was a new finding, which was advantageous to the secretion of intracellular protein. Farnesol-induced morphological transition is widely investigated in dimorphic fungi, but in the basidiomycete *C*. *versicolor* the morphological changes regulated by farnesol were also observed.

**Figure 3:**
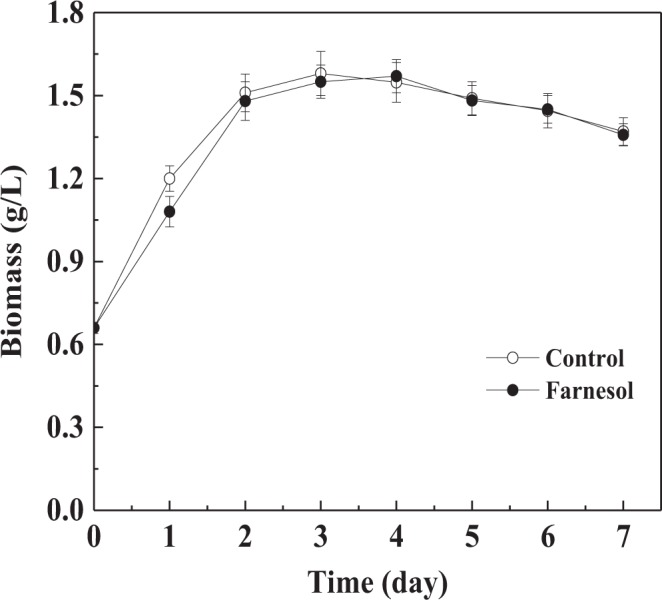
Effect of farnesol on cell growth during *C*. *versicolor* submerged cultures. The results are average of 3 replicate experiments. *Error bars* correspond to standard deviation.

In farnesol-induced cultures, *C*. *versicolor* exhibited the pelleted growth (smooth pellets). Mycelium biomass, expressed as grams (dry weight) per liter, achieved a peak value of 1.57 ± 0.06 g/L after 4 days of incubation (Fig. 3). No significant differences were observed between the farnesol-induced and control cultures that obtained a maximal value of 1.58 ± 0.08 g/L, indicating that *C*. *versicolor* cell growth was not affected by farnesol. It only changed the mycelial morphology of *C*. *versicolor*. This phenomenon was also observed in *O*. *piceae* liquid culture supplemented with farnesol^7^.

### Effect of farnesol on the relative expression levels of morphogenesis-related genes of *C*. *versicolor*

**Figure 4:**
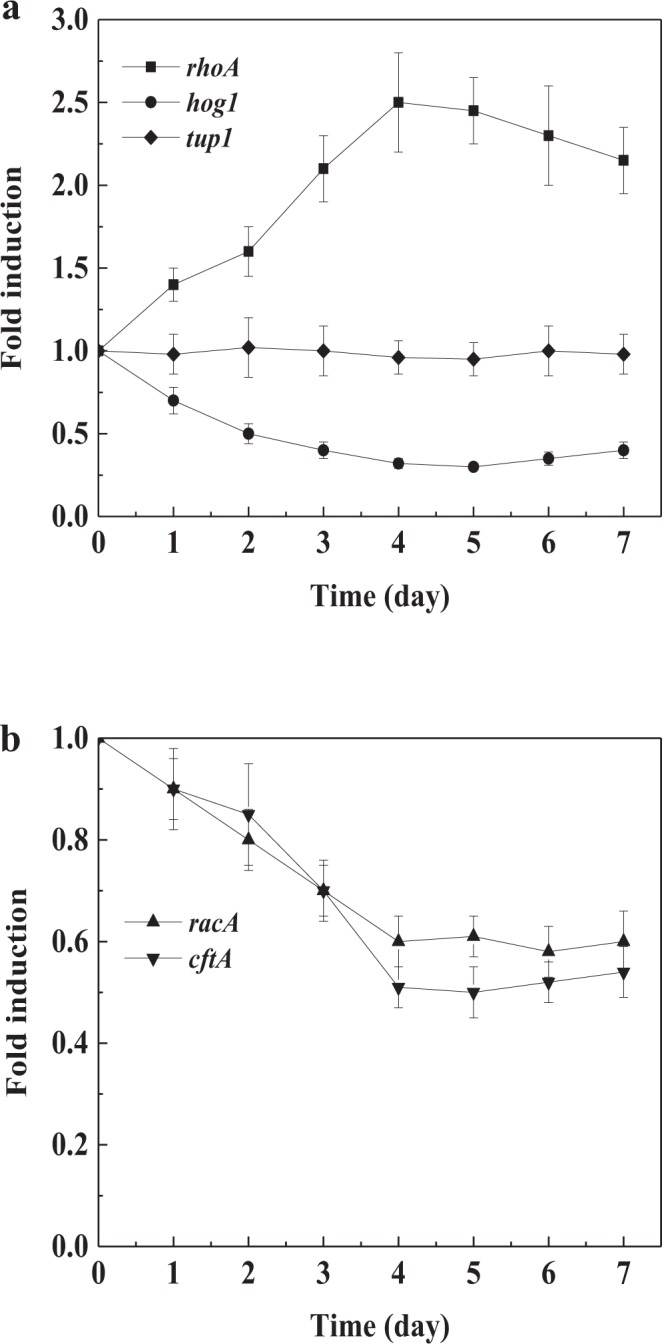
The relative expression levels of morphogenesis-related genes induced by farnesol during *C*. *versicolor* submerged cultures. (**a**) *rhoA*, *hog1* and *tup1*; (**b**) *racA* and *cftA*. The transcription levels of genes in farnesol-induced cultures were relative to that in control cultures without farnesol addition. The values represent the number of times each gene is expressed in induction group compared to control group (set at 1.0). Values are means of 3 replicates. *Error bars* correspond to standard deviation.

To understand how farnesol affected the morphology, the transcription levels of morphogenesis-related genes during culture process were analyzed using RT-qPCR assays. As shown in Fig. 4a, the relative expression level of *rhoA* in farnesol-induced cultures was gradually increased, and reached peak value of 2.5-fold compared with control cultures at day 4, when farnesol-induced mycelial morphology of *C*. *versicolor* was relatively distinct. Meanwhile, the transcription levels of *tup1* in the presence and absence of farnesol did not change significantly. Gene *hog1* in farnesol-induced cultures showed a relative expression of 0.3 times that of the control at day 4. Improved expression levels of *rhoA* meant that more polar growth sites were established, which then developed into new germ tubes and hyphae along the parental hyphae. In filamentous fungi, *rhoA* as a filament-inducing gene plays a central role in controlling polarity establishment and germ tube formation^13,35^. The expression of filamentous-suppressing gene *tup1* was not affected by farnesol, so that the filamentation gene *rhoA* was not suppressed. It is reported that *hog1* as a hyphal-suppressor gene regulates mycelial morphogenesis by inhibiting the expression of filamentation genes in Hog1 MAPK pathway in response to environmental stimuli^8,36^. Thus, the decrease of *hog1* expression level promoted the gene expression of *rhoA* through the signal pathway.

In addition, *racA* and *cftA* showed the relative expression of 0.6 and 0.5 times that of the control at day 4, respectively (Fig. 4b). Reduced expression levels of *racA* and *cftA* resulted in an actin localization defect, which made actin hyperpolarize, and thereby the loss of apical dominance and polarized tip extension. Moreover, actin hyperpolarization gave rise to frequent dichotomous branching phenotype with bulbous tips, which could be confirmed by Kwon *et al*.^13^. Gene *racA* and *cftA* maintain polarity apical dominance by regulating actin distribution at the hyphal apex^30,37^. Ras1-cAMP signaling pathway as a potential direct target for farnesol inhibition might be involved in decreasing the expression levels of *racA* and *cftA*^11,38^. Hence, it would be interesting to determine how farnesol affects the expression of morphogenesis-related genes through different signal pathways in future studies.

Together hyperbranched mycelial morphology with short hyphae and bulbous tips was developed from the synergistic effect of *rhoA*, *racA* and *cftA*. Additionally, Fig. 4 suggested that farnesol first regulated the expression of *rhoA*. After the formation of germ tubes and hyphae, farnesol then inhibited the expression of *racA* and *cftA*, and made the hyphae lose their polarized growth. The results were consistent with the functions of *rhoA*, *racA* and *cftA* during the growth and development of mycelium^13^. Gene *rhoA* has been identified to be associated with the formation of hyperbranched hyphae of *C*. *versicolor* in previous study^26^. But it was not elucidated that how farnesol affected the expression of *rhoA* gene. In this study, we pointed out that farnesol promoted the transcription of *rhoA* by down-regulating the expression of *hog1*. Furthermore, the down-regulation of *racA* and *cftA* expression was shown to result in the short hyphae with bulbous tips.

**Figure 5:**
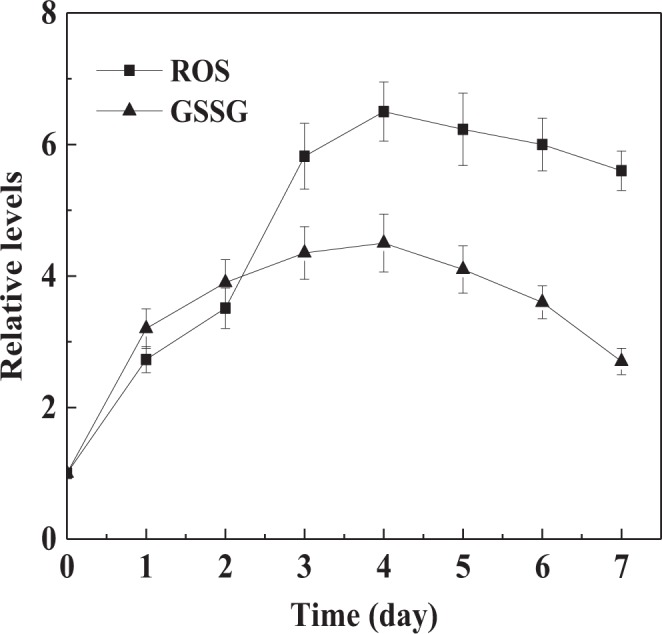
Time course of intracellular ROS and GSSG relative levels induced by farnesol during *C*. *versicolor* submerged cultures. The induction levels of ROS and GSSG in farnesol-induced cultures were relative to that in control cultures without farnesol addition. The values represent the number of times ROS and GSSG were induced compared to control group (set at 1.0). Values are means of 3 replicates. *Error bars* correspond to standard deviation.

Han *et al*. have reported that the oxidative stress is closely related to the morphogenesis of *C*. *albicans* by regulating the expression of filament-inducting genes. The transcription level of *hog1* can be down-regulated by enhanced oxidative stress in Hog1 MAPK pathway^8^. Some studies show that farnesol can trigger apoptosis of some pathogenic fungi by inducing mass synthesis of ROS^14,38^. In submerged processes of *C*. *versicolor*, we also observed that intracellular ROS was mass-induced upon the addition of farnesol into the cultures (Fig. 5). The accumulation level of ROS in farnesol-induced cultures was considerably more than that in control cultures. At day 4, the number of times ROS was induced reached the maximum of 6.5 compared with the control. Meanwhile, intracellular GSSG level was significantly enhanced by 3.5-fold relative to the control. The results suggested that a large amount of ROS was induced by farnesol that made the cells live in a physiological status with high levels of oxidative stress. Therefore, this high oxidative stress led to the decrease of *hog1* expression level that promoted the *rhoA* expression.

### Effect of farnesol on laccase production of *C*. *versicolor*

**Figure 6:**
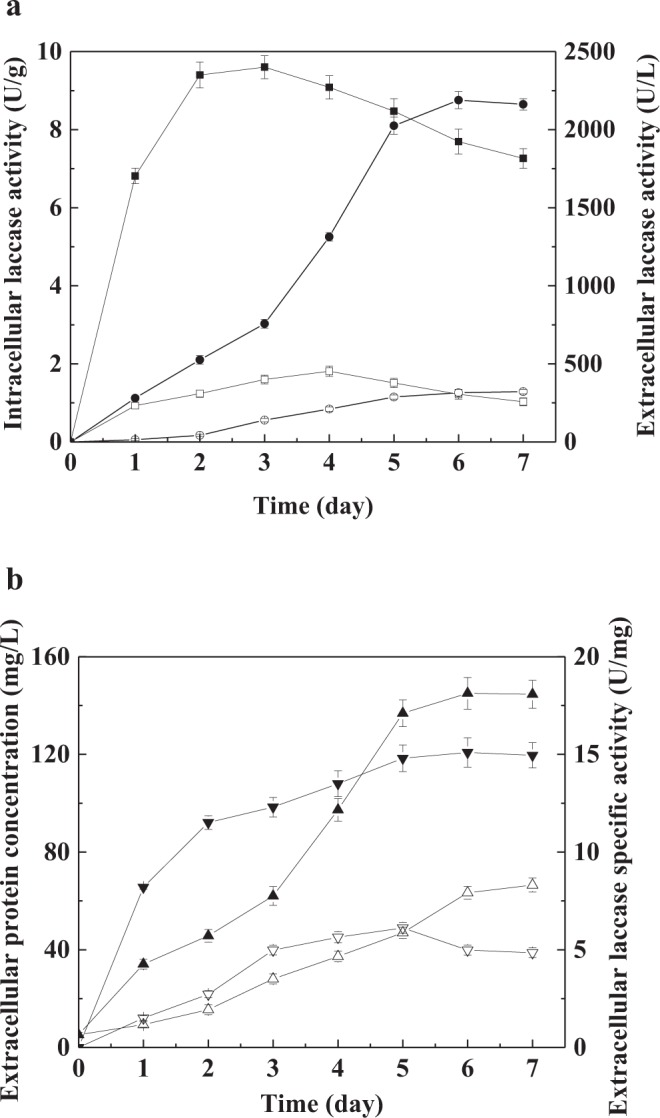
(**a**) Effect of farnesol on intracellular laccase activity and extracellular laccase activity during *C*. *versicolor* submerged cultures. Symbols: □, intracellular laccase activity (control); ■, intracellular laccase activity (farnesol); ○, extracellular laccase activity (control); ●, extracellular laccase activity (farnesol). (**b**) Effect of farnesol on extracellular protein concentration and extracellular laccase specific activity during *C*. *versicolor* submerged cultures. Symbols: Δ, extracellular protein concentration (control); ▲, extracellular protein concentration (farnesol); ∇, extracellular laccase specific activity (control); ▼, extracellular laccase specific activity (farnesol). The results are average of 3 replicate experiments. *Error bars* correspond to standard deviation.

*C*. *versicolor* as a white-rot basidiomycete can launch its own defense system and express large amounts of laccase to scavenge reactive oxygen free radicals^17,18^. This regulation happens at gene transcriptional level through antioxidant response element (ARE) that is differentially distributed in the promoter regions of laccase genes^23^. In farnesol-induced cultures, intracellular laccase activity increased up to 9.6 ± 0.3 U/g-biomass, which was much higher than that of the control (1.8 ± 0.1 U/g) (Fig. 6a). This showed that farnesol-induced oxidative stress response significantly stimulated the laccase biosynthesis.

Furthermore, the hyperbranched morphology with short hyphae and bulbous tips of *C*. *versicolor* induced by farnesol could further accelerate the secretion of intracellular laccase from cytoplasm to extracellular medium. The efficient secretion of laccase can relieve endoplasmic reticulum stress caused by mass-produced laccase, and thereby facilitate its correct expression and processing^2^. Consequently, extracellular laccase production in farnesol-induced cultures reached a maximum of 2189.2 ± 54.7 U/L at culture day 6, which was 6.8-fold greater than that of control cultures (322.5 ± 14.1 U/L) (Fig. 6a). Meanwhile, maximum extracellular protein concentration of 145 ± 6.5 mg/L was obtained around 144 h of incubation in the presence of farnesol, which was increased by 1.2-fold relative to the control (66.5 ± 2.8 mg/L) (Fig. 6b). The improvement of extracellular protein yield is due to increased intracellular protein secretion and laccase over-production^25^. Wongwicharn *et al*. reported that short hyphal elements with more branching were obtained by high levels of O_2_ enrichment in chemostat cultures of *A*. *niger*, which significantly increased extracellular glucoamylase production^39^. The effect of fungal mycelial morphology is rarely taken into account in the production of lignin-modifying enzymes including laccase. This finding is useful to the regulation of fungal growth and secretion of important enzymes of biotechnological interest by farnesol-regulated mycelial morphology.

**Figure 7:**
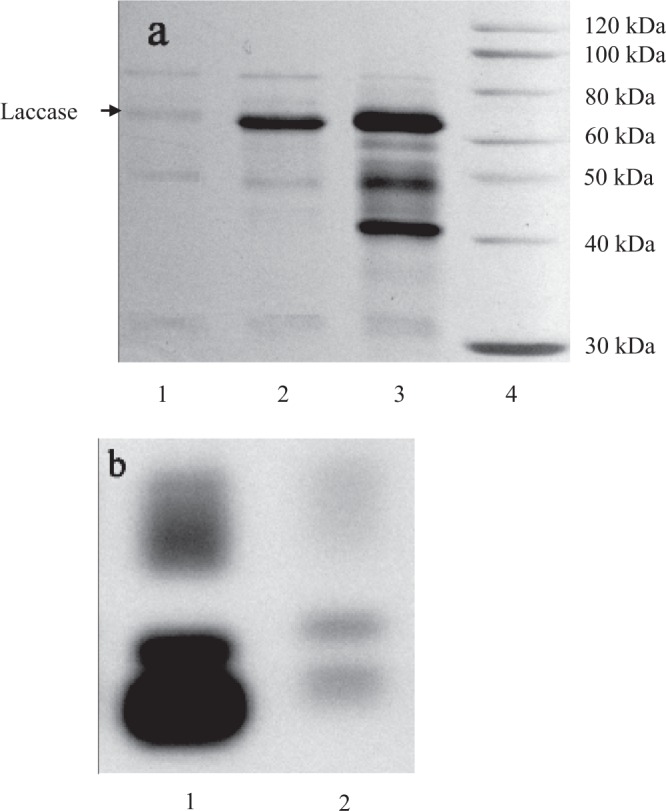
(**a**) SDS**-**PAGE pattern of extracellular culture supernatants (20 uL) with silver staining. Electrophoresis was carried out using a 12% cross-linked polyacrylamide gel. *Lane 1*: cell-free supernatants in control cultures; *Lane 2*: cell-free supernatants in farnesol-induced cultures; *Lane 3*: laccase standard from *C*. *versicolor* (Sigma); *Lane 4*: molecular mass marker proteins. (**b**) Native-PAGE pattern of extracellular culture supernatants (20 uL) with activity staining. After native-PAGE the laccase isozymes were stained with catechol. *Lane 1*: cell-free supernatants in farnesol-induced cultures; *Lane 2*: cell-free supernatants in control cultures.

In addition, extracellular laccase specific activity in farnesol-induced cultures reached to 15.1 ± 0.8 U/mg-protein that was 3.1 times that of control cultures (4.9 ± 0.3 U/mg) (Fig. 6b). As shown in Fig. 7a, farnesol significantly increased the proportion of laccase in the secreted extracellular proteins. Although other proteins were also increased, farnesol mainly promoted the production of laccase, which was advantageous to the separation and purification of laccase in downstream processing engineering. Three laccase isoforms were observed in the control and farnesol-induced cultures. The intensity of electrophoretic bands indicating the level of laccase isozymes biosynthesis in farnesol-induced cultures was significantly greater than that in control cultures (Fig. 7b). It showed that farnesol increased laccase production by promoting the expression of three isozymes.

**Figure 8:**
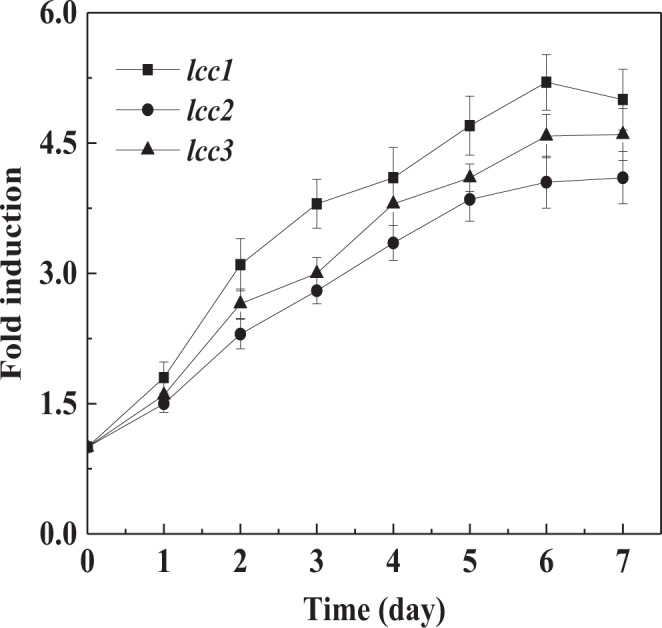
Time course of the relative expression levels of laccase genes (*lcc1*, *lcc2* and *lcc3*) induced by farnesol. The transcription levels of genes in farnesol-induced cultures were relative to that in control cultures without farnesol addition. The values represent the number of times each gene is expressed in induction group compared to control group (set at 1.0). Values are means of 3 replicates. *Error bars* correspond to standard deviation.

To understand how farnesol promoted the laccase biosynthesis, we investigated the effect of farnesol on transcription levels of laccase genes. *C*. *versicolor* genome encodes 10 putative laccase genes, but only three laccase isozymes are identified^31^. *lcc1*, *lcc2* and *lcc3* have been well characterized, so these three laccase genes are selected as the representative of gene expression levels. The transcriptional levels of *lcc1*, *lcc2* and *lcc3* during *C*. *versicolor* submerged cultures were analyzed using RT-qPCR assays. As shown in Fig. 8, the relative expression levels of *lcc1*, *lcc2* and *lcc3* were significantly induced by farnesol and gradually increased with incubation time, which reached the peak value of 5.2-fold, 4.1-fold and 4.6-fold compared with the control at culture day 6, respectively. Farnesol treatment resulted in a significant increase of intracellular ROS level in *C*. *versicolor* (Fig. 5), which would make the cells generate oxidative stress resistance. High oxidative stress could trigger the activation of nuclear transcription factors that traveled to the nucleus and bound to the ARE, thereby activating the promoter and inducing the transcription of laccase genes^23^. The elevated expression levels of laccase genes led to the increase of laccase biosynthesis to protect the cells from oxidative stress damage^18^. It is different from de Salas *et al*.’s results that higher sterol esterase activity is only attributed to higher proportion of mycelia and active hyphal tips in *O*. *piceae*, and its gene expression level is not affected by farnesol because esterase is not the oxidoreductase^7^.

Jaszek *et al*. have reported that paraquat dichloride (PQ) increases extracellular laccase activity as a part of oxidative stress response system. PQ is an ionic redox-cycling substance which catalyzes the overproduction of superoxide anion radicals and consequently other oxygen active species generated through Fenton-like reactions^17^. Farnesol is a chain-typed sesquiterpene alcohol^15^. Both PQ and farnesol promote laccase production by inducing ROS. The difference is that PQ inhibits the growth of *C*. *versicolor* and *Abortiporus biennis*^17^, but farnesol did not affect *C*. *versicolor* cell growth, probably because PQ is more toxic to cells. Then we investigated the effect of farnesol on laccase production from *P*. *sanguineus* and *T*. *pubescens* submerged cultures. As shown in Fig. S1, extracellular laccase activity in farnesol-induced cultures reached a maximum of 1860.8 ± 36.5 U/L for *P*. *sanguineus* and 985.2 ± 27.4 U/L for *P*. *sanguineus*, which were 6.0-fold and 4.5-fold greater than that of control cultures (308.4 ± 9.4 U/L and 217.3 ± 7.0 U/L). Together farnesol could effectively stimulate laccase production of several white rot basiodiomycetes (*C*. *versicolor*, *P*. *sanguineus* and *T*. *pubescens*), and especially the inductive effect on *C*. *versicolor* was stronger. We speculate that farnesol may have an induction effect on laccase and other oxidoreductases production from white rot fungi.

## Conclusions

Farnesol as a quorum sensing molecule in fungi was demonstrated to cause marked morphological changes of *C*. *versicolor* in submerged cultures. Farnesol was involved in regulating the expression of mycelial morphogenesis-related genes. Hyperbranched mycelial morphology with short hyphae and bulbous tips was developed from the synergistic effect of *rhoA*, *racA* and *cftA*, which was advantageous to the secretion of intracellular laccase. Extracellular laccase production was significantly promoted by farnesol, and its content in secreted extracellular proteins was greatly enhanced. The significant improvement of laccase production in farnesol-induced cultures was mainly attributed to increased expression levels of laccase genes, favorable pelleted growth, and highly branched hyphae with more bulbous tips that represented more protein secretion sites. This study provides a new insight into the mechanism for the enhancement of laccase production stimulated by farnesol. It also brings new opportunities in increasing the production of secreted enzymes (especially the oxidoreductases) of biotechnological interest in basidiomycetes.

## Electronic supplementary material

Supplementary Information

## Data Availability

All data generated or analyzed during this study are included in this published article and its Supplementary Information files.
