# Supplementary material for: Farnesol-induced hyperbranched morphology with short hyphae and bulbous tips of Coriolus versicolor
Source: Sci Rep. 2018 Oct 12;8:15213. doi: 10.1038/s41598-018-33435-6 (PMC6185903; doi:10.1038/s41598-018-33435-6)
Supplement: Supplementary file 1 — Supplementary Information [file 41598_2018_33435_MOESM1_ESM.docx]

*Article for Scientific Reports*

**Farnesol-induced hyperbranched morphology with short hyphae and bulbous tips of *Coriolus versicolor***

Ke-Feng Wang ^1, 2^, Chen Guo ^1, 2^, Fang Ju ^3 *^, Nadia A. Samak ^1, 2^, Guo-Qiang Zhuang ^4^, Chun-Zhao Liu ^1, 2, 5 *^

^1^ *State Key Laboratory of Biochemical Engineering, Institute of Process Engineering, Chinese Academy of Sciences, Beijing 100190, P.R. China*

^2^ *University of Chinese Academy of Sciences, Beijing 100049,* *P.R. China*

^3^ Department of Oncology, Qingdao Central Hospital, Qingdao 266042, P.R. China

^4^ *Research Center for Eco-Environmental Sciences, Chinese Academy of Sciences, Beijing 100085, P.R. China*

^5^ *Institute of Biochemical Engineering, Collaborative Innovation Center for Marine Biomass Fibers, Materials and Textiles of Shandong Province, School of Materials Science and Engineering, Qingdao University, Qingdao 266071, P.R. China*

***Corresponding author**

Dr. Chun-Zhao Liu, Institute of Process Engineering, Chinese Academy of Sciences, Beijing 100190, P.R. China, E-mail: czliu@ipe.ac.cn

Dr. Fang Ju, Department of Oncology, Qingdao Central Hospital, Qingdao 266042, P.R. China, E-mail: jufangjufang@sina.com

**Supplementary Information**

**Wang et al., Table S1**

**Table S1** Oligonucleotide primers used for RT-qPCR

| Primer name | Primer sequence | Gene specificity |
| --- | --- | --- |
| 18s-f | 5' GTAAAAGTCCTGGTTCCCC 3' | 18S |
| 18s-r | 5' CGATAACGAACGAGACCT 3' |  |
| rhoA-f | 5' GATGAGGATGACGTGGGAGTC 3' | *rhoA* |
| rhoA-r | 5'TGTAGAGGTCGATGGCAAGC 3' |  |
| racA-f | 5' CAAGCGTCTGGATGCTG 3' | *racA* |
| racA-r | 5' GACTGACGAGGGAGAAGCAG 3' |  |
| cftA-f | 5' GACGGACGTCTTCCTTGTCT 3' | *cftA* |
| cftA-r | 5' AAGATCGATTTGGGTGCCGA 3' |  |
| tup1-f | 5' CAAGGACTACGTGCTCTCCG 3' | *tup1* |
| tup1-r | 5' TTCTTGTGTCCCTGCAGCAT 3' |  |
| hog1-f | 5' GACGTCGTACTTTTGCCATGT 3' | *hog1* |
| hog1-r | 5' TTTGCGACTTTGGTCTCGC 3' |  |
| lcc1-f | 5' GCACGTTGATGATGAATCC 3' | *lcc1* |
| lcc1-r | 5' CGGTAGCGCTTGCCCTTC 3' |  |
| lcc2-f | 5' CAACTTCAACTTCACCATC 3' | *lcc2* |
| lcc2-r | 5' CTCGACATTGACCCATC 3' |  |
| lcc3-f | 5' CAGGGTCACACGATGAAC 3' | *lcc3* |
| lcc3-r | 5' CTCGACATTGACCCATC 3' |  |

**Wang et al., Fig. S1**

**a**

**b**

**Fig. S1** Effect of farnesol on extracellular laccase production during *P. sanguineus* **(a)** and *T. pubescens* **(b)** submerged cultures. The results are average of 3 replicate experiments. *Error bars* correspond to standard deviation.
